# 

**Published:** 1899-02

**Authors:** 


### MARRIAGES.

At Philadelphia, Pa., January 2, 1899, Dr. John J. Repp to Miss Miriam A. Colbert. Dr. Repp is a graduate of the Veterinary Department, University of Pennsylvania, and a member of the Pennsylvania and American Veterinary Medical Associations. He is now located at Ames, Iowa, having been selected for the Chair of Pathology and Therapeutics at the Iowa State Agricultural College, and Veterinarian to the Experiment Station, where the newly married couple will make their home after January 16th.

On December 22, 1898, Dr. J. W. Petty, of Winston, N. C., to Miss Eleanor Booker, Chapel Hill, N. C. Dr. Petty is the very efficient Secretary of the North Carolina Veterinary Medical Association.

---

Dr. Leonard Pearson spent several days in Harrisburg during the inaugural ceremonies of Governor Stone.

Dr. T. A. Geddes, of the Bureau of Animal Industry, recently located at Nashville, Tenn., has been transferred to departmental work at Washington, D. C.

